# Child witchcraft confessions as an idiom of distress in Sierra Leone; results of a rapid qualitative inquiry and recommendations for mental health interventions

**DOI:** 10.1186/s13034-021-00370-w

**Published:** 2021-04-09

**Authors:** Hélène N. C. Yoder, Joop T. V. M. de Jong, Wietse A. Tol, Joshua A. Duncan, Amjata Bayoh, Ria Reis

**Affiliations:** 1 University of Amsterdam, Amsterdam, The Netherlands; 2 Mental Health Coalition - Sierra Leone, Freetown, Sierra Leone

**Keywords:** Child Witchcraft, Child Idiom of Distress, Child and Adolescent Mental Health, Rapid Qualitative Inquiry, Sierra Leone

## Abstract

**Background:**

Reports about child witchcraft are not uncommon in sub-Saharan Africa. In this study we approach child witchcraft as an idiom of distress. In an environment that may prohibit children from openly expressing distress, the shared imagery of witchcraft can provide a cultural idiom to communicate about psychosocial suffering. We used an ecological approach to study how some children in distressing circumstances come to a witchcraft confession, with the aim to set out pathways for mental health interventions.

**Methods:**

We employed rapid qualitative inquiry methodology, with an inductive and iterative approach, combining emic and etic perspectives. We conducted 37 interviews and 12 focus group discussions with a total of 127 participants in Freetown, Sierra Leone. Inductive analysis was used to identify risk and protective factors related to witchcraft accusations and confessions.

**Results:**

We identified risk and protective factors related to the individual child, the family, peer relations, teachers and other professionals in a child’s life, traditional healers, pastors and the wider society. We found that in the context of a macrosystem that supports witchcraft, suspicions of witchcraft are formed at the mesosystem level, where actors from the microsystem interact with each other and the child. The involvement of a traditional healer or pastor often forms a tipping point that leads to a confession of witchcraft.

**Conclusions:**

Child witchcraft is an idiom of distress, not so much owned by the individual child as well as by the systems around the child. Mental health interventions should be systemic and multi-sectoral, to prevent accusations and confessions, and address the suffering of both the child and the systems surrounding the child. Interventions should be contextually relevant and service providers should be helped to address conscious and subconscious fears related to witchcraft. Beyond mental health interventions, advocacy, peacebuilding and legislation is needed to address the deeper systemic issues of poverty, conflict and abuse.

**Supplementary Information:**

The online version contains supplementary material available at 10.1186/s13034-021-00370-w.

## Background

Reports of child witchcraft accusations and confessions are not uncommon in Sierra Leone, a country of almost eight million people on the west coast of Africa.^1^ A local newspaper tells the story of a father who seeks justice for his children who were accused of witchcraft by his brother-in-law [1]. A Catholic priest writes about accused witches brought to the child care centre he leads [2]. Pastors, sometimes visiting from Ghana or Nigeria, address large crowds in the national stadium, with captivated audiences watching children confess to have caused accidents, sickness, and deaths [e.g. [3]]. The issue attracted the interest of the authors when doing research on child and adolescent mental health in the country [4]. We found that local explanatory models for child and adolescent mental health problems are mostly spiritual and may include involvement in witchcraft [4]. The relation between child witchcraft and child mental health has earlier been examined by Reis [5], who first described child witchcraft as an idiom of distress (IOD) [cf. [6]]. In line with earlier conceptualizations [7, 8], De Jong and Reis [9] define an IOD as *“an embodied symbolic language for psychosocial suffering that derives its legitimacy from its shared metaphors, meaning and understanding in a group”* [[9], p. 302]. An IOD allows individuals *“to express and communicate suffering caused by different types of stressors that cannot otherwise be expressed in the local social–cultural–political context, due to the inherent threat such expression would constitute to culturally dominant values and structures”* [[9], p. 302].

In this paper we approach child witchcraft confessions as an IOD. Children and families in Sierra Leone experience distress in many different forms. The country is still affected by the long-term consequences of the war that ended in 2002, both in terms of infrastructure (health, education) and intergenerational effects on mental health [10, 11]. According to the report on Multidimensional Child Poverty in Sierra Leone [12], up to 85.4% of children in Sierra Leone are deprived of one or more of the rights to shelter, education, information, water, sanitation, health or nutrition [12]. Adults and children questioned by Thulin et al. [13] listed multiple challenges at a personal or community level, including insufficient parental care, poverty, hunger, child labor, and a lack of access to health care. Children stated severe discipline or beating as their highest concern. A study by Zuilkowski et al. [14] confirmed that physical discipline is widely accepted and common in Sierra Leone.

Cultures differ in the way they respond to distress. An important notion in Sierra Leone is the ability to “bear” with difficult circumstances, or in the local Krio language *“bia”*, which the dictionary describes as *“endure, suffer patiently or resignedly, bear with”* [15]. The admonition to bear is commonly given to people who are sick, have lost loved ones, or experience other struggles in their lives. In her study of the Mende people of Sierra Leone (one of the largest ethnic groups), Bledsoe observed that parents considered the experience and endurance of hardship in childhood an important preparation for the future, enabling a person to bear with adversities in their adult life [16]. In addition, children hold a low status in Sierra Leone society [17] and are expected to be compliant and respectful of their elders [14]. These values and circumstances may well prohibit children from openly expressing their feelings related to distressing situations. However, children who grow up in an environment where belief in witchcraft is common may learn an alternative language [18] that enables them to express their distress in terms of the supernatural. Adults with these same ingrained values will likely accept a witchcraft narrative, especially when it offers an explanation for their own hardships as well. In this way child witchcraft confessions become an idiom of distress.

To better understand the context in which children confess to being witches it is important to note that belief in witchcraft is widespread across the world, including on the African continent [19]. Anthropologists have long abandoned the question whether witchcraft actually exists, and concluded that *“witchcraft exists as a social and cultural reality”* [[20], p. 6]. Witchcraft is a complex phenomenon with a broad variety of beliefs and practices across cultures [20], which continue to change over time [21, 22]. Currently, a fairly common notion of witchcraft shared across sub-Saharan Africa involves the belief in a mystical power known only to witches, which allows them to separate their souls from their physical bodies and enter into a spirit world from where they inflict harm on others [20, 23]. The beliefs about child witchcraft we will be addressing in this paper fall into this category. Whereas in the past it was assumed that belief in witchcraft would disappear under the influence of modernization and the growing influence of Christianity and Islam [20], contemporary social sciences emphasize that belief in witchcraft may well be part of a modernity and faith that are unique for Africa [21, 24]. The attraction of witchcraft is that it provides insight from the invisible world into the “why” of unfortunate events in the visible world [20], often furnishing answers that are lacking in modern science.

Sporadic accounts of children being accused of witchcraft in Africa date back as far as the 1930′s [25]. However, recent years have seen an apparent dramatic increase in reports of children who suffer from abuse resulting from witchcraft accusations, to the extent that it has caught the attention of various UN agencies and international NGOs [20, 21, 26, 27]. Reports of child witches are coming from both post-conflict and politically stable societies [5]. The question why children may have increasingly become accused of witchcraft remains largely unanswered [25]. Speaking about children in the Democratic Republic of Congo, De Boeck [22] describes the increased and prominent visibility of children in the urban landscape in a time of social and spiritual insecurity. With their potential for military and economic power and their defiance of traditional authority they are often considered a threat by older generations. While this may lead to witchcraft accusations, De Boeck suggests that *“the idiom of witchcraft […] also offers a possibility for children to challenge parents, public authority and the established order.”* [[22], p. 143]. The significant role of the African revivalist churches in the identification and treatment of child witches is frequently mentioned [21, 22, 25, 27–30]. Another important role is given to the media [25, 31], most notably Nigerian movies such as “End of the Wicked” [32], written by the influential Nigerian pastor Helen Ukpabio. Accusations of child witchcraft frequently lead to abandonment, lasting stigma and outright abuse [21, 31, 33].

Several authors have described common characteristics of child witches and witchcraft. The children often have distinguishing personal qualities such as a below or above average intelligence [5, 27, 33], or a chronic or life-threatening illness or disability [21, 23, 33]. They may have distinctive physical features [27], or display unusual behavior such as bedwetting, hyperactivity [28], precociousness, loquaciousness, speaking or acting like an adult and verbal or behavioral boldness towards adults [30]. Frequently these children are orphans or step children [21]. There are no accurate data related to gender [21] but accusations affect both boys and girls. With accusations also targeting infants and unborn children [30], age appears to be no limiting factor. Witchcraft can be passed on through lineage or through food that is offered to a child. Dreams play a significant role in the initiation [21, 23, 27]. Child witchcraft is more often seen in the context of poverty, but it is not the only contributing factor [22]. The witchcraft narratives told by child witches across countries show many similarities, and are often related to power-reversal, e.g.: children travel into the underworld^2^ where they can be adults, feast on human flesh and blood, own houses and cars, use ritual artefacts such as brooms and witch pots, change into dogs, cats or snakes, cause sickness and accidents, and make businesses fail [21, 27, 30].

These depictions however are limited. Not all child witches have deviating features. They often have only a lowly status in their family [25]. Many so-called characteristics of child witches are part of a child’s normal development [18]. Similarly, the distressing circumstances in which child witches may find themselves are not necessarily different from other children who don’t confess to be witches. This suggests that others factors are involved. In an anthropological study done for Unicef on contemporary witchcraft practice in Africa, Cimpric observed: *“Confession is still considered to be the most significant evidence in cases of witchcraft.”* [[21], p. 39]. This observation highlights the need to analyze the process that leads a child to a witchcraft confession. Rather than concentrating solely on individual child characteristics, we take in this paper the perspective of Bronfenbrenner’s ecological systems theory [34]. In his early work, Bronfenbrenner describes how children develop in a system of nested structures: (1) the “microsystem”: the immediate context of for example family, school and peers; (2) the “mesosystem”: the level at which the actors of the microsystem interact; (3) the “exosystem”: the settings in which a child is not present, but that have an influence on a child’s life, such as the parents’ work environment; and (4) the “macrosystem”: the culture or subculture in which the child grows up [34]. While Bronfenbrenner’s theory was further developed over the years, we limit our application in this paper to the central concept of nested environments. Within this ecological framework, we use the risk and protective factors approach, a research strategy commonly used in public mental health research to identify conditions that positively or negatively affect the chance that a condition develops [35]. Since intrapersonal characteristics of child witches do not seem to sufficiently explain child witchcraft confessions as an idiom of distress, we use the ecological approach to identify contextual and interpersonal risk and protective factors [cf. [35]]. In doing this, we can provide alternative explanations for the “evidence” of confessions and set out pathways for mental health interventions that prevent accusations and confessions and address the suffering that is expressed in the IOD.

## Methods

This research project is embedded in a wider investigation of child and adolescent mental health perceptions and systems of care in Sierra Leone [4, 36, 37], and an international research project on child IODs [5]. We employed rapid qualitative inquiry methodology, a time and cost-effective qualitative technique to gain information about a topic in a swift and concise way [38]. The approach was inductive and iterative, with initial data analysis (based on notes, observations and recollection of interviews and FGDs) taking place in team meetings during the data gathering, and emerging themes guiding consequent interactions with participants. Scientific rigor and validity of data was reached through triangulation (e.g. interviewing different participants related to one child or comparing general data obtained from different participants). The fieldwork took place over a period of two weeks in March 2014. The research team included two expatriate investigators, one of whom is a child mental health expert who at that time had lived in Sierra Leone for eleven years and who speaks the local language. They were complemented by a team of six research assistants from the area, who received a 10-h training before the rapid qualitative inquiry commenced, covering basic interview and FGD techniques and skills to deal with possible distress in participants. Bearing in mind the sensitivity of the research topic, ethical aspects of the research were reviewed using the Ethical Research Involving Children Compendium [39] and discussed in detail in a meeting with the Research Subcommittee of the Mental Health Coalition of Sierra Leone.^3^ The research proposal was approved by the Sierra Leone Ethics and Scientific Review Committee.

The sample included key informants (people with supposed knowledge about child witchcraft confessions in Sierra Leone) and purposively selected participants (identified or self-identified child witches, children and adults that directly related to them, and children and adults living or working in the communities where our research took place). They were identified through network sampling, whereby participants and research team members who were well-embedded in the local community were asked to suggest participants [40]. The sampling results were regularly discussed and evaluated in the research team. Decisions related to the inclusion of additional participants were based on data saturation, a need for additional information regarding a topic or a specific child, and the availability of participants. Table 1 gives an overview of the sample group (n = 127). In total we performed 15 child interviews, 10 interviews with people related to these children, 12 key informant interviews and 15 focus group discussions (see Additional file 1). One child interview had to be excluded due to poor recording quality.

**Table 1:** Research sample

	Male	Female	Total	Age range (years)	Average age (years)
Child participants					
Individuals	7	7	14	7–18	13.2
Focus group discussions [eight]	28	29	57	4–17	11.8
Subtotal children	35	36	71		

Adult participants					
Individuals*	6	3	9		
Focus group discussions [seven]	12	23	35		
Key informants	11	1	12		
Subtotal adults	29	27	56		
Total participants**	64	63	127		

*Caregivers, pastors and teachers, interviewed in relation to specific children, plus a child who in the interview revealed he was actually 25 years old

**A few individuals/key informants were also participants in the focus group discussions

Data gathering took place through semi-structured or unstructured individual interviews and focus group discussions (FGDs). Interviews with individual children who were personally associated with witchcraft (either through accusation or confession) involved questions about demographics, stressors (current and past) and related feelings, thoughts, behavior and relations, and the witchcraft accusation and/or confession (see Topic List in Additional file 2). Adults around them (caregivers/parents, teachers, pastors) were asked open questions about the child and his/her behavior and relationships, and the witchcraft accusation or confession. The protocol for the FGDs was applied with flexibility. A vignette was provided (see Additional file 3) with a story based on familiar Sierra Leonean child witchcraft stories. Interviewers frequently made use of this vignette, but in other cases chose other ways to introduce the topic, for example by first discussing other problems related to children, or even introducing the topic of witchcraft quite directly, followed by open questions about the information that was provided. Similarly, the approach to key informant interviews was flexible. In most cases, the key informants were comfortable talking about the topic of witchcraft which then could be presented quite directly. In other cases, key informants were first asked to give their opinion on other problems that affect children (identified by the research team as common problems, such as maltreatment or sickness), after which the topic of witchcraft was introduced. Open questions were asked in response to answers given. Except for team meetings and a few key informant interviews with individuals who spoke English, all interviews and FGDs took place in Krio, the lingua franca of Sierra Leone. Verbal informed consent was retrieved prior to the interview or FGD.

The research took place in Freetown, the capital of Sierra Leone, with a centrally located, impoverished slum community as its point of entrance, while subsequent sampling led the team to various other (lower-middle class) areas, including shelters for vulnerable children, and a church-run school in a suburban area bordering Freetown. All interviews, FGDs and team meetings were recorded, and notes were taken for the clarification of recordings where needed. Since English and Krio are closely related, the majority of interviews were translated during transcription by the first author who speaks Krio, with the exception of relevant idioms, which were first transcribed in Krio and then translated into English. The FGDs were transcribed in Krio and translated into English through hired services.

Inductive analysis was used to identify factors leading to witchcraft confessions [41]. The first author read through all the materials and extracted relevant data in an Excel spreadsheet that allowed analysis on different levels of Bronfenbrenner’s socio-ecological system, e.g. information related to the family, peers and school that are part of a child’s microsystem; information on religious leaders that are commonly part of a child’s exosystem, and societal beliefs that form part of the macrosystem. The first and last author developed a coding system to identify data related to (1) stressors, (2) child emotions, (3) child behavior, (4) child perceptions and cognitions related to witchcraft, (5) adult perceptions and cognitions related to witchcraft, (6) confessions, and (7) risk and protective factors for witchcraft accusations or confessions. Data related to individual stories of children confessing to witchcraft were compared with the general data. The results were then discussed with all authors for further analysis. The various professional (psychiatry, psychology, public mental health, clinical child & family studies, medical anthropology and sociology) and cultural (Sierra Leonean and Dutch) backgrounds of the authors provided multi-disciplinary and intercultural perspectives.

## Results

### Introduction

While studying the phenomenon of child witchcraft we came across various other spiritual problems affecting children, most commonly “having eyes” (the ability to see in the spiritual realm), seeing devils, and marriages to “night husbands or wives” (spirits or demons). While these conditions are of concern, our participants made it clear that they are distinctly different from child witchcraft. In this paper we therefore limit ourselves to children confessing to witchcraft. This reduced our number of individual case studies from fourteen to nine children who were accused of practicing witchcraft, of which five children also confessed.

Our Results section is organized as follows. First, we present a case study that relates the story of “Tamba” (pseudonym), one of the children we interviewed. We chose this story not because it was unique (we heard many similar stories), but because it was the most complete narrative related to one child, with access to multiple actors. The story was reconstructed by the first and last author based on interviews with Tamba himself, his foster mother, his cousin/classmate, his teacher and a pastor related to the school.

The Case Study is followed by four sections that describe findings related to child witchcraft as an idiom of distress: (1) stressors faced by children and adults in Sierra Leone; (2) children’s emotional/behavioral responses to distress; (3) witchcraft beliefs that provide the language to communicate about distress; and (4) socio-ecological dynamics of accusations and confessions. After this we present our findings related to risk and protective factors for witchcraft confessions, both at the child’s personal level and in the widening socio-ecological systems: family, peers, child professionals (teachers and social workers), healers, pastors and society.

### Case study: “Tamba”

When Tamba was 20 months old, his father – who lives abroad – gave him to his older sister to raise. Tamba is now nine years old and still living with his paternal aunt (hereafter referred to as his foster mom), who is childless. She has recently entered menopause and seems to be grieving over the fact that she has lost the chance to ever give birth to a child of her own. He says he is treated well by her family. It is not unusual for children in Sierra Leone to be raised by their relatives, and as is common in these situations, Tamba got to spend a recent vacation with his birth mother who lives about ten miles down the road. While there, he sustains an injury to his hand which leads to prolonged and costly treatment. During the same vacation, Aminata, a stepsister of his mother (sharing the same father but not the same mother) gives Tamba a meal containing meat. Shortly after this, Tamba has a disturbing dream. He cannot remember the details, but when he tells his mother about it, she concludes that Aminata – whom she believes to be a witch – is about to initiate Tamba into witchcraft. Tamba’s mother takes him to the traditional healer to obtain protection from being initiated. However, Aminata begins to appear to him in his dreams. She orders him to destroy his father, mother and extended family, but Tamba refuses because he knows his parents support him financially. Consequently, he falls sick. In the following weeks and months he experiences stomach aches, vomiting, dizziness and malaria, causing his family to now spend a lot of money on treatments from healers.

After the Christmas holidays Tamba returns to his foster mom. There he tells one of his cousins, a girl who is also his classmate, that he is a witch. He tells her to keep it a secret. “He was bluffing”, she later tells us. His foster mom notices that since his return he is more “stubborn” in his behavior. Others observe that Tamba starts spending more time away from home, and that he does not seem to be afraid of anybody. Back in school, two young teachers with little teaching experience are taking care of Tamba’s class. One of them describes Tamba as a troublesome boy who mocks him behind his back. The teacher reprimands Tamba, but does not seem to be free from fear. In our interview with him he narrates how he told Tamba: “My friend, the way you are behaving, it’s like you are a witch. (…) Let me tell you, if you are demon-possessed, you won’t be able to do anything to harm me.” On another occasion Tamba and some of his friends get in trouble over some video games that are brought to school. Unacceptable behavior in Tamba’s classroom is met with various forms of discipline, most frequently flogging.

One day, as Tamba shares some juice with his friends during lunch time, the teacher asks him where he got the juice. After Tamba refuses to answer, the teacher stretches him on a table and flogs him 24 times. The chronology is not completely clear here but around this time a pastor who is related to the school (and who believes he is called by God to deliver children from evil) speaks to the students and invites anyone involved in witchcraft to come forward. He warns that anyone who does not respond will die within a week. Tamba is one of eight children, four boys and four girls, who respond to the call. The four boys are the same boys who got in conflict with the teacher about the video games. In the lengthy confessions the different roles of the children in the witch realm are explored, and Tamba is identified as the one who initiated all the other students. This theory seems to be accepted by all involved, even though Tamba tells us later he had no idea that the other children who went forward were witches. In fact, up till then, he himself had not believed that he was a witch. The students confess that they are weary of the teachers’ discipline and frequently report the teachers to the “Mamie Queen”, their leader in the witchcraft realm, in hope that she will take revenge. At one point during confession Tamba attracts attention when he shows off his supernatural skills by “stealing” money out of a teacher’s pocket. While nobody denies that the money never physically left the teacher’s pocket, all believe that Tamba supernaturally took it and, after being ordered to do so, placed it back.

Tamba’s mother, grandmother and foster mom are invited to the school where they receive the news that Tamba has confessed to have brought calamity on the family: he claims to have caused his foster mom’s recent accident and taken both her and his mother’s money to the underworld by having them spend it on hospital bills for him and another child.

Tamba impressed the research team as a shy boy, who sometimes seems hesitant to talk but repeats multiple times that Aminata initiated him. Although he usually feels happy, he feels sad and ashamed that people believe he practices witchcraft.

### Stressors

Both children and adults mentioned multiple stressors in the lives of children in Sierra Leone, and more specifically, their personal living situations. Inductive analysis revealed two broad categories: stressors related to poverty and stressors related to tensions in relationships. Poverty affects children’s housing, nutrition, education, basic resources such as clothing, and access to healthcare.

“We are hungry when we come to school. (…). And our teachers find it difficult to teach us. They teach us, but they don’t pay them. (Primary school student who is aware that many teachers go without salaries).

The effects of poverty are exacerbated by natural phenomena such as the heavy rains that affect the country every year during the rainy season and that cause an increase in deaths from malaria and other diseases.^4^ Participants from one community mentioned the problem of flooding. The decreased life expectancy and high child mortality rate that often accompany poverty were reflected in the frequent mentioning of early deaths of parents and siblings. It is important to note that all these stressors affect not just the children, but the systems around them as well. As illustrated in the quote above, teachers in Sierra Leone may receive minimal or no wages. A healer described how mothers can be gone from their homes all day to earn an amount barely enough to feed their family. One key informant with multiple wives had lost seven of his twenty children.

Relational stressors that were mentioned included complicated relationships between children and stepparents, conflicts within families, severe physical discipline, abuse and neglect. Relationships with teachers can become a source of distress when children are forced to pay teachers in order to pass their tests, or when physical discipline is severe.

### Emotional and behavioral responses to stressors

When asked about feelings children can have in relation to stressors, children mentioned sadness, anger, fear, loneliness, and disappointment. In one of the FGDs children explicitly denied anger as an emotion in response to injustice. Adults responded that in distressing circumstances children may experience sadness, envy of others, a lack of peace in their heart, hatred, inner conflict, etc. In a discussion around a vignette some children acknowledged negative feelings but also immediately stressed the need to bear with an abusive situation in hopes that things will get better.

Both children and adults understood the causal connection between the feelings of a child and his/her behavior. Former homeless children frequently mentioned that negative feelings would make them go out to the street, sometimes accompanied by stealing. Others mentioned cursing, being stubborn, disobedient and wayward, but also withdrawn or absent-minded (thinking about other things). One participant observed:

“… if you are staying in a place where you do not want to live, the tendency is there to do some bad things that will make them associate you with witchcraft.”

### Witchcraft beliefs

Children grow up with witchcraft beliefs around them. Witchcraft confessions are public; they take place in the community or in public places such as the chief’s compound or a church. Adult participants generally freely shared their beliefs, opinions and experiences. It was obvious in the FGDs that children are very familiar with witchcraft narratives. With minimal prompting they told multiple stories of child witches in their homes, schools and communities. Children learn from Nigerian witchcraft movies too and may not always interpret these movies as purely fictional.

Despite the participants’ vast knowledge of witchcraft beliefs and narratives, and their confident descriptions of child witch characteristics, adults frequently emphasized that they would not be able to determine whether a child is a witch or not. Some of the children had learned to show a similar reluctance. Only traditional healers or pastors have the skills and authority to give a verdict on a witchcraft accusation. While people may express suspicions, any other person who takes authority in this regard is suspected to have supernatural powers or even be a witch themselves.

Some traditional healers we spoke to believed child witchcraft is increasing in Sierra Leone, and attributed it to the high population growth and intergenerational tensions:

“… as the number of children they give birth to is increasing, so the witchcraft increases (…) the system they grow up with, compared to the one we grew up with, is very different. (…) at that time, we feared our mothers, we feared our fathers. But now, there is no fear.”^5^

### Socio-ecological dynamics of accusations and confessions

Witchcraft accusations set in motion social and psychological processes that are challenging to reverse. Participants made it clear that there are virtually no confessions without an accusation. Once an accusation is made, the pressure on a child is immense. Wanting to avoid the impression of being able to determine whether someone is a witch or not (and thus being an accomplice in witchcraft), it is likely that nobody will stand up for the child. In response to a vignette, a social worker described how a child will feel after an accusation:

“He is always absent minded. He is always thinking about himself. How would he be able to protect himself? Because he already has it at the back of his mind that there is no security for him here: ‘There is nobody who will provide security for me.’ So he always has that thinking.”

The distress and consequent behavior of the child may subsequently intensify the suspicion of witchcraft.

A child was suspected of killing her foster mother. When she was questioned about it, she started stammering. This made the accusers call the traditional healer who confirmed she was the witch.

Children who confess to be witches may do this for different reasons. Many participants believed that the threatening and intimidating circumstances in which the accusation is made make children confess. One child told us he only confessed to protect his aunt (it was not clear from what, but possibly it was a fine). Another child said he confessed to avoid further abuse by his mother and stepfather. Two children were called witches by their peers and decided to agree in an effort to stop the bullying. The seven children confessing alongside Tamba may have responded to the threat that children who did not confess would die within a week. Some children genuinely believe they are witches. One of them was a 10-year-old boy who impressed us as so depressed and traumatized that we had to avoid probing and keep our interview short.

Children who confess are commonly questioned about who initiated them, who they may have initiated and who else they have met in the witchcraft realm. By identifying other children, grandmothers, aunts or neighbors, their confessions create turmoil in families and communities and perpetuate the omnipresent belief in witchcraft practices.

Although we did not formally investigate what happens after a confession is made, we were told multiple stories of lasting stigma, school dropouts and of children being returned to their families in the provinces.

### Risk & protective factors for witchcraft accusations and confessions

#### Individual

Given the wide variety of child witch characteristics, it is almost impossible to identify specific individual characteristics that put a child at risk of being accused of and/or confessing to witchcraft. Our impression is rather that accusations and confessions take place in the context of life events (especially the occurrence of misfortunes) and interactions with or within the surrounding systems (e.g., a child’s disobedience; a pastor hinting at witchcraft as a cause for structural tensions in polygamous or blended families).

“He [the child accused of witchcraft] won’t confess. Except if something happens with the parents or the guardian, something extraordinary happens in the house, for example there is hardship in the house.” (White Garment Church leader).

That being said, characteristics that most frequently were named as typical features of a child witch were being bold, outspoken, stubborn, not afraid of anything, and especially not afraid of adults. Other suspicious behaviors may be stealing, lying, frequently breaking things, being very quiet and withdrawn, or not fulfilling social expectations such as not crying when a relative or close acquaintance dies. Some participants described girls behaving like women, or children who are frequently sick. Some of the physical characteristics seem to indicate poverty or neglect: children who are skinny, dirty, or who wear clothes that don’t fit. An important characteristic witnessed in school is that the child cannot concentrate and does not perform well academically. Dreams play a significant role in the identification of child witches, especially dreams in which the child consumes food. In an environment where food is often scarce, these dreams are likely common, but in combination with other factors they may lead a child to believe that they have been initiated into witchcraft.

A child dreams that a friend offers him food. In church the next Sunday, the pastor identifies him as a witch. The boy does not believe him, but since he had the dream, he accepts the allegation, as does his family.

Children are aware that dreams will frequently be interpreted in terms of witchcraft. Some may therefore choose not to share their dreams. This discernment could potentially be considered a protective factor.

#### Family

Although participants said that witchcraft accusations can be made by biological parents, we did not see much evidence of this. Rather, participants frequently mentioned an increased risk of witchcraft accusations for children who do not live with their biological parents, especially children who come from the provinces where witchcraft is believed to be rampant. Of the nine children we spoke to who were accused of witchcraft, only two were accused and/or confessed while they lived with one of their biological parents.

Some mothers we spoke to differentiated between witch accusations and name-calling, a rather commonly used method to stop undesired behavior. While in the view of the parent this may be effective, we do not know if children are always able to make the distinction between serious accusations or name-calling in relationships with people of authority. This practice can thus become a risk factor.

Families can play a protective role when people outside the family accuse a child of being a witch. A mother advised her son not to respond to others calling him witch:

“But my mother told me not to say anything. God for sure knows that I am not a witch. So she says that I should just let them talk.” (Former homeless child)

When children are believed to be falsely accused of witchcraft, families will usually defend them. They may take the accusation to the local chief to vindicate the child. One father successfully stood up for his son who was called a witch and subsequently expelled from school. When the suspicion or accusation is raised within the biological family or the family raising the child, the caregivers usually seek counsel with a traditional healer or pastor. As we will see below, this may increase the risk of an accusation or confession.

#### Peer relations

Children talk among themselves about witches. In the case study we see how Tamba boasts about being a witch, even though he apparently does not (yet) really believe he is one. Children may put themselves at risk with this behavior.

A child tells her friends she wants to kill her teachers. Shortly after this she “changes into a snake” [the description suggests she may have had a seizure]. This prompts the other students to tell the teachers what the girl has said. The head mistress expels the child from school.

Accusations may lead to bullying, and the child’s response may only confirm the suspicion.

A young boy in a village is accused of being a witch. Back in school the other students start provoking him. The boy pushes one of them over, who consequently breaks his arm. This makes the teachers conclude he must be a witch. The boy is expelled from school.

As we saw in the case study, sharing food with friends – a very common and appreciated habit in Sierra Leone – can become a reason for suspicion. Teachers told us that even sharing pens, pencils or erasers could be a way of initiating others, making sharing practices a risk factor.

#### Teachers and other professionals

Our sample included two groups of people who relate to children professionally: teachers and social workers. A teacher of Tamba’s school (see case study) told us: *“… there are other pupils in this school that are demon-possessed. In fact, most of them [pupils in the school] are children of the night.”* As we saw in the case study, the suggestion of being a witch was first made by Tamba’s teacher. Teachers of another school seemed to have a different mindset. They did not deny the existence of child witchcraft but said they had never encountered it in their school. They also considered other explanations for deviant behavior.

“… as a teacher you will look at the psychological behavior of that child. (…) if a child comes into the class, if they come up with a snake movie, then we can normally ask: ‘Why?’ (…) In our own world, you observe maybe their mother, it is domestic harassment or it is hunger that is affecting them.”

One key informant who doubled as a pastor and teacher told us how he had handled a situation in his classroom with both a traditional, spiritual and psychological approach. Nobody was blamed and peace returned to the classroom. Contrarily, at another school a girl dreamt that someone was trying to offer her food. Her father made a complaint to the teacher, a pastor was consulted and a child was identified and forced to confess. Knowledge about child development and mental health in professionals interacting with children can be a protective factor. A lack of training can be a risk, but so can be the fear that affects professionals. A social worker was only willing to relate to a child accused of witchcraft after he promised not to harm her. Despite her willingness to help, she corroborated the witchcraft allegation.

#### Healers

Traditional healers play a significant role in the lives of children accused of witchcraft. Healers may perform with their devil mask (“ariͻgbo”) in local communities and actively find witches. They are also consulted by families or caregivers who suspect a child of witchcraft. When a chief is asked to rule in a witchcraft case, he may invite traditional healers to give a verdict on the witchcraft accusation. The relationship with traditional healers is complicated and ambivalent. Children expressed extreme fear of them:

“I panic, I tremble everywhere when I am close to them.” (Teenage boy, former homeless child)

Children can easily be intimidated by the fearful looking costumes and ceremonies. They may not understand what is going on and answer questions just to show their submission to the adult:

“He may not even [know] what the implication is; he just says, ‘Yes, Sir.’” (Social Worker).

Adults also report discomfort with traditional healers and their role in the community. They are considered both powerful and manipulative. People depend on healers to cure diseases, offer protection from witchcraft, predict misfortune and prescribe ways to prevent it. However, both adults and children acknowledge that the healers have a strong economic interest in their witch finding activities. Traditional healers are believed to have power to make people say or do things against their will and in some cases even kill people with their ceremonies. Once a traditional healer becomes involved and even more if he expresses the verdict of witchcraft, it becomes almost impossible for a child to withstand the accusation.

“So there is no way out again because they say, ‘anything that a “Mͻreman” [Muslim diviner or healer] says, is final.’ So I just have to accept, because they say I am a witch. I just have to bear the punishment.” (Former homeless child in FGD).

One social worker observed that children who confessed during public witch finding ceremonies often had nothing to say anymore once the witch finders had left. There were a few stories where accusations of children were not confirmed by healers. However, we do not know enough about the dynamics around these cases to know what the interests of the healer could have been. In one situation, a healer diagnosed an alternative spiritual condition that was less stigmatizing but still would require money to be healed. In another situation, a boy was vindicated of a witchcraft accusation but instead a girl of the same household was accused and made to confess. Healers often live in the community they are serving. They may be familiar with structural and temporary tensions in the family and the community, and thus seek to manipulate the dynamics of the context around a child. We did not see evidence of healers ever questioning the witchcraft narrative. Their strong financial interest in the outcome of the process is a risk factor for accusations.

#### Pastors

With the growth of Pentecostal churches in Sierra Leone, pastors have increasingly become important actors in witchcraft allegations. Vulnerable or sensitive children attending emotionally charged worship services with charismatic pastors may feel a need to come forward when claims are made about the presence of child witches. Attendance of these services can be a risk factor for these children.

“Even this last one, that Apostle Suleiman from Nigeria came into Sierra Leone, so many little, little, little children came out (…) running, 'I am a witch, I am a witch'.” (Teacher).

As illustrated in the case study, the invitation to confess may be accompanied by threats against those who do not confess. Some churches have a stronger emphasis on beliefs in witchcraft than others, but those who do seem to be gaining more popularity. Families may no longer always feel comfortable with traditional healers but still want help for their child.

“… they [the churches] bring temporal respite (…) if not for the victims, but for their families. (…) our children, no more we do believe in this traditional approach, but our children need some attention. And there is a religious entity that is willing to give us this attention; we are willing to take them there.” (Sociologist).

Pastors who strongly believe in witchcraft may not question the witchcraft narrative that a family presents to them.

Compared to traditional healers, participants spoke less of possible financial gain for pastors, although one participant implied it by condemning religion as a pathway to making money. It seemed however that the status acquired by delivering witches is just as important. Similar to traditional healers, the involvement of a pastor can become a risk factor for a child suspected of witchcraft. The story of the pastor/teacher who dealt successfully with a witchcraft allegation in his classroom shows that this does not always have to be the case. His knowledge of child development and mental health in addition to his theological training may have made the difference here.

#### Society

Children in Sierra Leone grow up in a society where witchcraft narratives are widely accepted. None of our participants seemed to deny the presence of witchcraft in society and the involvement of children in it. These beliefs are strong. Even within the research team it was hard to have some people look at child witchcraft from an alternative perspective. After working together for two weeks, meeting frequently to discuss our findings and possible alternative interpretations for witchcraft confessions of children, one of our research team members announced his final conclusion about the children we interviewed regarding their alleged or confessed involvement in witchcraft: *“They are all witches.”*

Because of their position in society, children are vulnerable and an easy target for witch finders. *“… how many children will be given the permission to defend themselves? And even if they defend themselves, what is the place of children in our society that people could believe them?” (Sociologist).*

Children themselves realize that they are often being taken advantage of:

“… because they have power over us, the little ones, that makes that they always abuse us.” (Primary School Student, FGD)

At the community level, the chiefs traditionally rule in witchcraft cases. They can play a protective role, as was illustrated in a story where a chief ordered traditional healers to release a child held captive on accusation of witchcraft.

## Discussion

### General observations

As Friedman & Howie observed in their study of the Salem Witch Trials of 1692/1693 [42] and Roper in his study of the Augsburg Witch Craze of 1723 [43], we found that very few people in Sierra Leone question the witchcraft narratives, since the stories of the children are generally in line with common beliefs about witchcraft. The absence of clear boundaries between the physical and supernatural world [44] makes children’s experiences in the witch realm fully plausible [cf. [45]]. While the Western research team members were searching for psychological or material benefits that could explain why children would be attracted to witchcraft, participants were not disturbed by the fact that the perceived benefits were all in the supernatural realm. Because these children are believed to have real power and benefits, they are also not looked upon as children to be pitied but as children to be feared [cf. [21, 22]]. However, it is important to note that any assumed power and benefits seem to exist only in the perceptions of people around the child. Being a “witch” is ultimately not an empowering experience for children, who rather respond with bewilderment at the thought of practicing witchcraft.

From a mental health perspective, the recently published study by Thulin et al. [13] on cultural concepts of distress among children in Sierra Leone sheds an interesting light on our findings. Many of the indicators of distress described in this study are similar to the child witch characteristics listed by our participants.^6^ Providing a contextually relevant vocabulary for mental health symptoms in children through the definition of cultural concepts of distress may be a first step in de-mystifying the behavior of children who are thought to be witches.

### Child witchcraft as an idiom of distress

Our findings suggest that child witchcraft as an IOD, in the systemically oriented definition of Reis and De Jong cited above, is not so much owned by the individual child as well as by the systems around the child. In many cases, the child could be considered the “Identified Patient” whose behavior is a manifestation of problems or stressors in the surrounding systems [cf. [5]]. Cultural values (such as the importance of being able to “bear with” circumstances) may prohibit adults and children from openly expressing distress related to family tensions, conflicts, poverty, etc., but it is acceptable to ascribe these issues to witchcraft and to discuss the stressors in that context. In this way witchcraft enables communication about distress. Within this process, children are vulnerable; they cannot stand up for themselves in accusations. Consequently, they may outwardly accept the allegation of being a witch, and inwardly either accept or discard the notion. Their confessions seem to be made mostly for pragmatic reasons such as avoidance of trouble and abuse, peer pressure, or protection of relatives. However, in this paper we look beyond the directly observable explanations for a confession to the deeper issues that cause people to use the witchcraft idiom.

As for the systems around the child, De Boeck already mentioned the importance of acknowledging the genuine fear that adults have of child witches and the undeniable risk of being named as a witch by a child who confesses [22]. Our results also point to the perception of cultural decline: adults believing that the current generation of children does not show respect for their parents anymore [cf. [14]]. Apart from these fears and perceptions, our study shows that it is just as important to acknowledge the actual suffering in the systems around the child: parents struggling with financial constraints, conflicts and losses, peers struggling with poverty and oppressive systems, teachers struggling with minimal resources, etc.

### The process leading to confessions

**Fig. 1:**
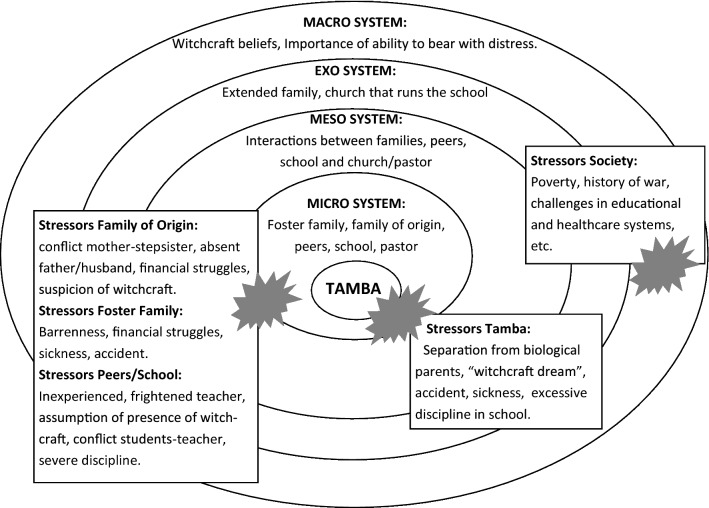
Bronfenbrenner’s ecological systems applied to case study

To understand the process leading to confessions we will first consider the Case Study presented above. Figure 1 illustrates how Tamba’s life is embedded in the systems around him. As a 9-year-old child, his microsystem includes his foster family, his family of origin, his peers and his school. The adults and children in Tamba’s microsystem experience multiple stressors, such as financial constraints, conflicts, health-related problems, severe discipline, etc. Tamba also experiences distress on a personal level: he is separated from his biological parents, he injures his hand, has distressing dreams and frequently is unwell. At the mesosystem level, various actors in the microsystem interact and potentially influence the developmental pathway of a child [35]. In this case, we see how the mesosystem reinforces the witchcraft narrative, e.g. the worries of Tamba’s mother and the suspicion of the teacher are confirmed by the pastor; the cousin who considered Tamba a friend and did not seem concerned about his witchcraft stories now learns in school that he is a witch. Usually there is no direct contact between the child and its exosystem, and for Tamba this is probably the case with his extended family and the church that runs the school. Important aspects at this level are the apparent tensions in his mother’s family and the fact that the church running the school is an indigenous church with a strong emphasis on supernatural healing and deliverance from evil forces. All Tamba’s systems are embedded in a society (macrosystem) where witchcraft is a common explanatory model and where the ability to bear with distress is considered an important value. Although not explicitly mentioned by Tamba or his family, like all families in Sierra Leone, they are affected by nation-wide stressors such as poverty, a recent history of war, and an under-resourced health and educational system.

The Case Study of Tamba describes the accumulating events, beliefs and suspicions within the various systems that ultimately lead Tamba to confess. The tipping point [46] in this process seems to be the pastor’s challenge and threat directed at the children, but his teacher also plays a pivotal role as he expresses suspicions and severely disciplines Tamba not long before his confession. We see how Tamba’s confession brings relief and/or affirmation to the systems around him: Tamba’s mother is justified in her belief that her stepsister is a witch, Tamba’s stepmother has an explanation for the misfortunes of her family, Tamba’s peers are excused for their troublesome behavior as they were unknowingly initiated by him into the underworld, and Tamba’s teacher has a reason for his inability to maintain order in the classroom. Even the pastor is confirmed in his identity of being called by God to bring deliverance to children affected by evil forces. While there were brief moments when Tamba and his friends seemed to be empowered by the witchcraft narrative and used it against their teachers, in the end we are left with a sad and ashamed 9-year-old boy. As Reis observed earlier: *“whereas witchcraft idioms may offer a healing resource at the group level by scapegoating and exorcising evil, this destroys the accused child.”* [[5], p. 635].

The process leading to Tamba’s confession is not unique. While individual stories will vary, in each situation there will be risk and protective factors that influence the process, and a tipping point that turns the course of events towards a confession or away from it. In the results we have mentioned some of these risk factors but also the protective roles that families and teachers can play when they have a better understanding of child development, age-appropriate behavior, and common responses to distress. We see that the tipping point often is related to the involvement of a religious authority figure who rarely questions the narrative and has a vested interest in the outcome. In the few cases where witchcraft is denied by the religious leader, it is not uncommon to see people from the surrounding systems continue their search for help until their suspicion is confirmed or an alternative solution is offered.

### Recommendations for interventions

In this section we highlight some recommendations to consider in the development of interventions that prevent child witchcraft accusations and confessions, and address the distress that is expressed in child witchcraft as an IOD.

1. Since the tipping point for an accusation or confession is often related to the moment a traditional healer or pastor gets involved, it could be proposed that interventions should be primarily aimed at attitudinal and behavioral change of the religious leaders. This may not be an easy task as they are the same people who propagate belief in child witchcraft. Some people have argued that it is not the belief in witchcraft that is harmful, but the actions based on that belief [21, 23]. However, as Briggs and Whittaker argue, this *“position is problematic as, in affording toleration of beliefs, the causal connection between the application of beliefs and child abuse is avoided.”* [[47], p. 2163] A focus on “changing the way child witches are treated” perpetuates the belief in child witchcraft with all its consequences. Challenging the belief system is an important religious effort that needs to come from within local societies to prevent it from being dismissed as another “neo-colonial” imposition [31]. Efforts towards this have been made by Christian organizations in other parts of Africa who question the theological validity of local concepts of witchcraft [48–50].
2. Since our findings suggest that the decision that a child is a witch is usually made at the mesosystem level (where actors of the microsystem level interact), mental health interventions should be systemic (including the family) and inter-sectoral (e.g. targeting the community and school), a recommendation which is supported by general guidelines for child and adolescent mental health care [51]. Despite the reservations we just expressed, religious leaders can be considered (non-professional) community-based practitioners of mental health care [37] and therefore should be included in these interventions. Contextually relevant education on child development and mental health, and an understanding of the connection between psychosocial suffering and witchcraft narratives may create alternative understandings of witchcraft confessions and prevent witchcraft accusations. Training of teachers should also include classroom and behavioral management, while parenting skills training may reduce abusive parenting practices and help prevent escalations at the family level [52]. Since living with people other than the biological parents can be a risk factor for child witchcraft accusations, the common practice of sending children away to live with others should be reevaluated and regulated from a child protection perspective.
3. Since child witchcraft as an idiom of distress is owned by the systems around the child, interventions will need to go beyond the needs of the individual child and address the very real distress of those around the child. Safe and appropriate alternatives to cope with stressors need to be offered. Before “Western” coping strategies are promoted, local helpful and acceptable means of resilience will need to be explored and developed into meaningful interventions [53].
4. Since witchcraft beliefs run deep, capacity building should be an important component of the dissemination of any intervention [37, 54]. Service providers need to be supported to acknowledge their fears of witchcraft and learn how these affect their interactions with accused or professing child witches. Trainers from a Western background need to be willing to scrutinize their own worldview and be receptive to a worldview that accepts witchcraft as a reality [55]. Approaching the children’s witchcraft narratives as childhood fantasies [cf. [43]] may be an interesting and possibly relevant academic perspective, but will at this point most likely not be effective in a society where witchcraft is accepted across social strata.
5. Beyond mental health interventions, advocacy, peacebuilding and legislation is needed to address the deeper systemic issues of poverty, conflict and abuse.

### Limitations

Our study was limited by the fact that our research team had limited experience in qualitative research. Training and supervision were provided, but were restricted by time. The quality of data collection was monitored, but we also had to accept that our self-funded research could not afford highly educated research assistants. The challenges of capacity building in the educational context of Sierra Leone have been described earlier in a paper by the first author [37]. The topic of witchcraft resonated with conscious and subconscious beliefs and fears in the research team members, and fascination with the witchcraft narrative sometimes affected data gathering when more attention was given to the content of the narratives than the human dynamics surrounding them. Access to children identifying as witches was complicated by the hesitation of participants to point them out. However, we believe that the use of triangulation, in-depth interviews and a multidisciplinary approach, in the context of the wider research in which this study is embedded, has increased the validity of our findings. Data gathering for this paper took place in 2014, but the authors who continued living in Sierra Leone (both in Freetown and in the rural areas) have continued to encounter cases of child witchcraft accusations and confessions.

This study took place in the urban setting of Freetown, and although many of our participants frequently referred to experiences they have had while living or spending time in other parts of Sierra Leone, caution should be practiced in extrapolating the results to the rest of the country, especially the rural areas.

## Conclusions

In this paper we describe child witchcraft confessions as an Idiom of Distress primarily owned by the systems around the child. The ecological approach made it possible to tease apart the different parts of the process leading from distress on multiple levels to a child witchcraft confession. This study confirms the need to challenge harmful beliefs and practices related to child witchcraft, but also highlights the importance of interventions that address the very real suffering of not only the child but also the systems around it.

## Declarations

## Supplementary Information

**Additional file 1.** Overview FGD participants

**Additional file 2.** Topic list interview child

**Additional file 3.** Vignette

## Data Availability

The datasets used and/or analyzed during the current study are available from the corresponding author upon reasonable request.

## Abbreviations

FGD: Focus Group Discussion
IOD: Idiom of Distress

## Footnotes

1 For a historical and cultural background on Sierra Leone in the context of mental health, see Yoder-van den Brink [37].

2 In Sierra Leone, the term “underworld” is used to describe the realm of spirits and witches.

3 This meeting took place on Friday November 22, 2013 and was attended by 8 members of the subcommittee and coalition.

4 Traditional healers in one community attributed the increase in deaths during the rainy season to an increase in witchcraft activities, since the heavy rains interrupt the usual witch finding operations.

5 The word “fear” in Krio has a connotation of both fear and reverence or respect.

6 This applies especially to indicators related to the cultural concepts of “gbos gbos”, “poil at”, “diskoraj” and “wondri”. Although not studied in-depth, the indicators for “fred fred” seem similar to the spiritual condition of “having eyes”.
