# Supplementary material for: Child witchcraft confessions as an idiom of distress in Sierra Leone; results of a rapid qualitative inquiry and recommendations for mental health interventions
Source: Child Adolesc Psychiatry Ment Health. 2021 Apr 9;15:18. doi: 10.1186/s13034-021-00370-w (PMC8035751; doi:10.1186/s13034-021-00370-w)
Supplement: Supplementary file 1 — Additional file 1. Overview FGD participants [file 13034_2021_370_MOESM1_ESM.pdf]

FGD Adults		Male	Female	Total
FGD 1	Community Elders	4	3	7
FGD 3	Social Workers Child Shelter 1	0	3	3
FGD 8	Community Members	1	3	4
FGD 9	Social Workers Child Shelter 2	3	1	4
FGD 12	Mothers	0	6	6
FGD 14	Teachers School 1	2	3	5
FGD 15	Teachers School 2	2	4	6
	**TOTAL**	12	23	35

FGD Children		Male	Female	Total	Average Age
FGD 2	Boys in Shelter	6	0	6	14.8
FGD 4	Students	3	4	7	10
FGD 5	Students	1	3	4	14.5
FGD 6	Students	2	3	5	13.4
FGD 7	Students	6	4	10	10.2
FGD 10	Boys in Shelter	6	0	6	12.3
FGD 11	Girls in Shelter	0	9	9	9.3
FGD 13	Students	4	6	10	12.8
	**TOTAL**	28	29	57	
