# Supplementary material for: Child witchcraft confessions as an idiom of distress in Sierra Leone; results of a rapid qualitative inquiry and recommendations for mental health interventions
Source: Child Adolesc Psychiatry Ment Health. 2021 Apr 9;15:18. doi: 10.1186/s13034-021-00370-w (PMC8035751; doi:10.1186/s13034-021-00370-w)
Supplement: Supplementary file 2 — Additional file 2. Topic list interview child [file 13034_2021_370_MOESM2_ESM.pdf]

VIGNETTE FOCUS GROUP DISCUSSION - BOYS

Alimami, a 13-year boy from Kono, is living with his uncle and aunt and their children in Freetown. When they brought him from upcountry, his aunt and uncle promised to send him to school, but this hasn't happened. While his cousins go to school, Alimami is working around the house, cleaning and fetching water. Later in the day he goes out and sells "cold water" for his aunt. He usually eats the leftovers of his cousins' food.

How do you think Alimami feels? How may this make him behave?

Have you ever had these feelings? What do you do when you have these feelings?

Alimami does not get along well with his youngest cousin. The boy, who is younger than him, often provokes him. But he never gets punished, while Alimami often gets beaten by his uncle. Then, one day his youngest cousin gets very sick and dies.

How do you think Alimami feels about the death of his cousin?

How do you think he will behave?

Alimami's aunt is very upset about the death of her child. She tells her husband that she is sure that Alimami is a witch and has killed her son. Soon everyone seems to believe that Alimami is a witch. Alimami denies: he is not a witch and he did not kill his cousin.

How do you think Alimami feels now that everyone calls him a witch?

Do you think Alimami is a witch? Why or why not?

Alimami's aunt takes him to traditional healer. The healer asks him many questions. Finally Alimami confesses: he is a witch and he killed his cousin.

Why do you think Alimami confessed?

Do you think he is a witch? If yes, why? If not, why not?

Do you know any children who have had a similar experience?

(Is there a chance we can talk to them?)

VIGNETTE FOCUS GROUP DISCUSSION - GIRLS

Aminata, a 12-year girl from Kono, is living with her uncle and aunt and their children in Freetown. When they brought her from upcountry, her aunt and uncle promised to send her to school, but this hasn't happened. While her cousins go to school, Aminata is working around the house, cleaning and fetching water. Later in the day she goes out and sells "cold water" for her aunt. She usually eats the leftovers of her cousins' food.

How do you think Aminata feels? How may this make her behave?

Have you ever had these feelings? What do you do when you have these feelings?

Aminata does not get along at all with her youngest cousin. The boy, who is younger than her, often provokes her. But he never gets punished, while Aminata often gets beaten by her aunt. Then, one day her youngest cousin gets very sick and dies.

How do you think Aminata feels about the death of her cousin?

How do you think she will behave?

Aminata's aunt is very upset about the death of her child. She tells her husband that she is sure that Aminata is a witch and has killed her son. Soon everyone seems to believe that Aminata is a witch. Aminata denies: she is not a witch and she did not kill her cousin.

How do you think Aminata feels now that everyone calls her a witch?

Do you think Aminata is a witch? Why or why not?

Aminata's aunt takes her a traditional healer. The healer asks her many questions. Finally Aminata confesses: she is a witch and she killed her cousin.

Why do you think Aminata confessed?

Do you think she is a witch? If yes, why? If not, why not?

Do you know any children who have had a similar experience?

(Is there a chance we can talk to them?)
