# Supplementary material for: Child witchcraft confessions as an idiom of distress in Sierra Leone; results of a rapid qualitative inquiry and recommendations for mental health interventions
Source: Child Adolesc Psychiatry Ment Health. 2021 Apr 9;15:18. doi: 10.1186/s13034-021-00370-w (PMC8035751; doi:10.1186/s13034-021-00370-w)
Supplement: Supplementary file 3 — Additional file 3. Vignette [file 13034_2021_370_MOESM3_ESM.pdf]

CIOD RESEARCH – INTERVIEW INDIVIDUAL CHILD

SOCIO-DEMOGRAPHICS

Name:	Age:	Sex:
Family Composition:		
Birth Place / Place of Origin:		
Year of Arrival in Freetown (if applicable):	Current Living Area:	
Religion:	Ethnic Group:	
Highest Completed Level of Education:		

TOPIC LIST INTERVIEW

□ Problems (focus on <u>relevant</u> issues)

□ Effect on Self

□ Emotions
e.g.
- How do you feel? (probe for specific feelings)
- Where are these feelings located in your body?

□ Thinking
e.g.
- What do you think about…
- What do you think is the cause of …
- What is your major fear about…

□ Behaviour
e.g.
- What do you do when you feel…
- How do you cope with / solve …

□ Relation to / Effect on other children

□ Relation to/ Effect on Family

Notes:

Notes (continued):

Date: Name Interviewer(s):
